# Draft Genome Sequence of *Corynebacterium pseudotuberculosis* Strain PA05 Isolated from an Ovine Host in Pará State, Brazil

**DOI:** 10.1128/genomeA.00082-17

**Published:** 2017-03-30

**Authors:** Alyne Cristina Sodré Lima, Vitória Almeida Gonçalves de Moura, Kenny da Costa Pinheiro, Carla Thais Moreira Paixão, Wana Lailan Oliveira da Costa, Adriana Ribeiro Carneiro Folador, Ana Luiza de Mattos Guaraldi, Rommel T. J. Ramos, Artur Silva, Joana Montezano Marques

**Affiliations:** a Laboratory of Genomic and Bioinformatics, Federal University of Pará, Center of Genomics and System Biology, Belém, Pará, Brazil; b College of Medical Science, State University of Rio de Janeiro, Rio de Janeiro, Brazil

## Abstract

We report here the draft genome sequence of *Corynebacterium pseudotuberculosis* PA05, isolated from an ovine host in Pará State, Brazil. *C. pseudotuberculosis* is an etiological agent of diseases with veterinary and medical importance. The genome contains 2,435,137 bp, a G+C content of 52.2%, 2,295 coding sequences, five pseudogenes, 53 tRNAs, and six rRNAs.

## GENOME ANNOUNCEMENT

*Corynebacterium pseudotuberculosis* is a Gram-positive, nonmotile, nonsporulated, pleomorfic, intracellular, and aerobic-facultative bacterium (1). *C. pseudotuberculosis* is mainly known as the etiologic agent of caseous lymphadenitis (CLA), a chronic disease in small ruminants (goats and sheep) and is also considered an important agent to other diseases such as ulcerative lymphangitis and ulcerative dermatitis, among others. This pathogen affects several species, including sheep, goat, horse, cattle, llama, alpaca, buffalo, and human (2).

CLA is a contagious disease that is characterized by the presence of abscesses in the lymph nodes and internal organs of the host. After infection, the bacterium becomes encapsulated within walled-off lesions from which they escape immune system-mediated action, initiating a state of persistence (3). The infection presents itself as a disease difficult to eradicate. It is responsible for large economic losses resulting from the damage it inflicts on livestock, such as reducing the quality of wool, decreasing milk production, lowering animal weight, and causing death and damage to carcasses (4). The bacterium has numerous survival mechanisms and uses many strategies to adapt to its environment; the two toxic factors most described in the literature are a lipid factor that improves cellular wall resistance against digestion by cellular enzymes and exotoxin with hemolytic capacity that increases vascular permeability and facilitates invasion (5). Here, we report the genome sequencing of *C. pseudotuberculosis* PA05, a strain isolated from a punctured caseous abscess located in the lymph nodes in the posterior thigh region of a sheep host (Santa Inês breed) in Pará, Brazil.

The genome was sequenced with the Ion Torrent PGM platform using a fragment library that generated 1,251,198 reads. FastQC software (http://www.bioinformatics.babraham.ac.uk/projects/fastqc) was used to evaluate the quality of the reads, and the FASTX toolkit (http://hannonlab.cshl.edu/fastx_toolkit) was used to perform filtering and trimming, in which reads with a Phred quality score of ≥ 20 were removed. The trimmed reads were then assembled using SPAdes version 3.9.0 (http://bioinf.spbau.ru/spades) with *k*-mers 31, 33, 35, 61, 63, and 65. The assembly generated 24 contigs. The number of contigs was reduced to eight using the Lasergene 11 Core Suite tool (http://www.dnastar.com/t-dnastar-lasergene.aspx). The scaffold was generated using Mauve version 2.4.0 (6) with *C. pseudotuberculosis* strain 1002B (CP012837.1) as the reference genome. Gap closure was done using GapBlaster (7) and CLC Genomics Workbench (http://www.clcbio.com). The final scaffolding with six contigs was submitted to the RAST (Rapid Annotations using Subsystems Technology) server (http://rast.nmpdr.org) for automatic annotation. The draft genome has 2,435,137 bp, 2,295 coding sequences, a G+C content of 52.2%, five pseudogenes, 53 tRNAs genes, and six rRNAs genes.

### Accession number(s).

This whole-genome shotgun project has been deposited in GenBank under the accession number CP019159.
